# Optimization of cervical cord synthetic T _1_ -weighted MRI for enhancing clinical application in neurodegenerative spinal cord disorders

**DOI:** 10.1162/imag_a_00225

**Published:** 2024-07-15

**Authors:** Simon Schading-Sassenhausen, Maryam Seif, Nikolaus Weiskopf, Patrick Freund

**Affiliations:** Spinal Cord Injury Center, Balgrist University Hospital, University of Zurich, Zurich, Switzerland; Department of Neurophysics, Max Planck Institute for Human Cognitive and Brain Sciences, Leipzig, Germany; Felix Bloch Institute for Solid State Physics, Faculty of Physics and Earth Sciences, Leipzig University, Leipzig, Germany; Wellcome Centre for Human Neuroimaging, UCL Queen Square Institute of Neurology, University College London, London, United Kingdom

**Keywords:** synthetic T _1_ -weighted MRI, quantitative MRI, accuracy, neurodegeneration, spinal cord injury

## Abstract

Synthetic MRI offers the advantage of reducing acquisition time and enhancing flexibility through the reconstruction of various contrast weightings from a single set of MRI scans. However, the use of synthetic T_1_-weighted (synT_1_-w) MRI can lead to potentially biased measurements of the cross-sectional area (CSA) in the spinal cord when compared to conventionally acquired T_1_-weighted MRI. This disparity can have implications for comparability and sensitivity of MRI in assessing disease progression or treatment effects in neurodegenerative spinal cord disorders. Thus, this study aimed at improving the accuracy (i.e., difference between synthetic and acquired MRI) of cervical cord CSA measurements (C1-C3 level) based on synT_1_-w MRI implementing a longitudinal data set acquired from 23 acute spinal cord injury (SCI) patients and 21 healthy controls over 2 years. Moreover, the validity of using synT_1_-w MRI for tracking cervical cord atrophy following SCI over 2 years was verified. SynT_1_-w images were reconstructed from quantitative maps of proton density, longitudinal, and effective transverse relaxation rates derived from a multi-parameter mapping protocol. The results showed a minimal bias of -0.31 mm^2^(-0.5%) in CSA measurements based on synT_1_-w compared to acquired MRI. Estimates of atrophy rates and average CSA were comparable between synthetic and acquired MRI. A sample size estimation for detecting treatment effects on CSA atrophy after 2 years following SCI revealed that the required sample size is reduced by 13.5% using synT_1_-w instead of acquired MRI. This study shows high accuracy of synT_1_-w MRI and demonstrates its applicability in clinical studies for optimizing long MRI protocols.

## 1 Introduction

T_1_-weighted magnetic resonance imaging (T_1_-w MRI) such as 3D Magnetization Prepared Rapid Acquisition Gradient-Echo (MPRAGE) is routinely used for imaging the central nervous system in both research and clinical practice. It is an established method to assess macrostructural changes in tissue structure such as regional brain atrophy and changes in cross-sectional area (CSA) of the spinal cord (Chien et al., 2020;Cohen-Adad et al., 2021;Freund et al., 2010;Seif et al., 2019). Macrostructural changes of the spinal cord are commonly seen in various diseases, including spinal cord injury (SCI) and multiple sclerosis, and were suggested as potential biomarkers for clinical outcomes (Casserly et al., 2018;Seif et al., 2019).

In the clinical domain, acquisition time is a critical factor that significantly impacts patient comfort, the quality of scans, and the occurrence of artifacts. Thus, prolonged scanning protocols can compromise the overall image quality and subsequent diagnostic accuracy (Oztek et al., 2020). One solution for reducing acquisition time is synthetic MRI, since various contrast weightings can be flexibly estimated from quantitative MRI of physical tissue properties (Callaghan et al., 2015;Nöth et al., 2015;Tanenbaum et al., 2017).

Two important performance measures for imaging methods are accuracy and precision. While accuracy refers to the difference between the measured value and the ground truth, the precision describes how close repeated measurements are (i.e., repeatability). Both parameters are important measures for clinical studies. Previously, we investigated the precision of synthetic T_1_-w (synT_1_-w) MRI for assessing CSA of the upper cervical cord and for tracking neurodegeneration following SCI (Schading, Seif, et al., 2023). The synT_1_-w MRI was reconstructed based on the quantitative multi-parameter mapping (MPM) protocol (Helms et al., 2008;Weiskopf et al., 2013). This study showed high test-retest repeatability both within the same scanner as well as across different MRI scanners and vendors, which opens the opportunity of applying synthetic MRI in multi-center studies. However, the absolute estimates of CSA derived from synT_1_-w showed still a remaining systematic bias of -1.59 mm^2^(-2.5%) compared to the reference method MPRAGE, which affects the direct comparability of the results with studies using the MPRAGE standard (Chien et al., 2020;Cohen-Adad et al., 2021;Freund et al., 2010;Lundell et al., 2011). Moreover, for the application of synT_1_-w-derived measures as predictive biomarkers in clinical routine, highly accurate estimates of these markers are required. This is particularly important for biomarkers derived from a single measurement, which would be advantageous for patient benefit, as opposed to biomarkers requiring repetitive assessments (Kirshblum et al., 2016). Therefore, decreasing the bias of CSA measurements based on synT_1_-w compared to the established MPRAGE sequence (i.e., increase the accuracy with respect to MPRAGE) would improve the comparability and facilitate the implementation of synthetic MRI in clinical routine.

The aim of this study, therefore, was (1) to further increase the accuracy of synT_1_-w MRI by optimizing the reconstruction parameters for minimizing the bias in CSA (i.e., difference between synT_1_-w and the reference standard MPRAGE), (2) to validate the optimized synT_1_-w images for tracking atrophy in the cervical cord following SCI, and (3) to compare the required sample sizes for detecting potential treatment effects on cervical cord atrophy between MPRAGE and synT_1_-w.

## 2 Methods

### 2.1 Participants and study design

This retrospective study is based on the same longitudinal data set as in our previous study (Schading, Seif, et al., 2023) and includes 23 patients with acute SCI (male (m)/female (f): 17/6, age [mean ± SD]: 46.8 ± 19.2 years), who were consecutively admitted to the rehabilitation program at the Balgrist University Hospital between September 2010 and August 2014, and 21 healthy controls (m/f: 13/8, age [mean ± SD]: 33.7 ± 9.8 years) (Azzarito et al., 2020;Freund et al., 2013;Grabher et al., 2015;Schading, David, et al., 2023;Seif, Curt, et al., 2018;Seif, Ziegler, et al., 2018;Ziegler et al., 2018). On the American Spinal Injury Association Impairment Scale (AIS), 8 patients were initially rated as AIS A, 5 as AIS B, 3 as AIS C, and 7 as AIS D. The initial neurological level of injury ranged from C3 to T11 (see (Schading, Seif, et al., 2023) for a comprehensive overview of clinical information). Both healthy controls and SCI patients underwent MR imaging at five different time points—shortly after injury/inclusion in the study (baseline measurement), 2 months, 6 months, 12 months, and 24 months following enrollment/SCI. In patients, the mean (±standard deviation [SD]) interval from the date of injury to the first scan was 1.5 (±0.5) months, to the second scan was 3.8 (±1.5) months, to the third scan was 7.3 (±2.0) months, to the fourth 13.8 (±3.6) months, and to the fifth 28.3 (±4.3) months. Both groups differed with respect to age (p = 0.017, Wilcoxon rank-sum test), while no sex imbalance was evident (p = 0.52, Fisher’s exact test).

### 2.2 Ethics statement

All participants provided written informed consent before participating in the study, which was approved by the local Ethics Committee of Zurich (EK-2010-0271).

### 2.3 Image acquisition

Participants underwent the following MRI scans: a T_1_-weighted 3D MPRAGE and the quantitative MPM protocol on clinical 3T scanners (Siemens Verio and Siemens Skyra, Erlangen, Germany). The field of view (FoV) of both protocols covered the brain and the cervical cord approximately from C1-C5. The following parameters for the MPRAGE sequence were used: 176 slices, in-plane FoV: 224 × 256 mm^2^, 1 mm^3^isotropic resolution, repetition time (TR) = 2420 ms, echo time (TE) = 4.18 ms, flip angle = 9°, inversion time (TI) = 960 ms, readout bandwidth: 150 Hz/pixel, and total scan time: 9:04 min. The MPM protocol consisted of three 3D multi-echo fast low-angle shot (FLASH) gradient-echo sequences each with different contrast weightings (T_1_-, proton-density (PD)-, and magnetization-transfer (MT)-weighted). The commonly shared sequence parameters were as follows: 176 slices, FoV: 240 x 256 mm^2^, 1 mm^3^isotropic resolution, parallel imaging (GRAPPA) with a speedup factor of 2 in the phase-encoding direction (anterior-posterior), partial Fourier acquisition with a 6/8 sampling factor in the partition direction (left-right), and readout bandwidth: 480 Hz/pixel. TR of T_1_-, PD-, and MT-weighted contrasts were 25 ms, 25 ms, and 37 ms, respectively; the flip angles of T_1_-, PD-, and MT-weighted contrasts were 23°, 4°, and 9°, respectively. Six (MT-weighted) and eight (PD- and T_1_-weighted) echoes with equidistant echo spacing of 2.46 ms were acquired with the first TE being 2.46 ms. The total acquisition time was 23 minutes.

### 2.4 Calculation of synthetic T _1_ -weighted MRI

First, MT-w, PD-w, and T_1_-w echoes were co-registered to account for possible subject motion between the echoes. Next, quantitative maps of PD, R_1_, and R_2_* were generated based on T_1_-, PD-, and MT-weighted images using the “Create hMRI maps” tool of the hMRI toolbox (v0.2.0) (Tabelow et al., 2019) within the SPM12 (v7487) environment. RF transmit field B_1_^+^inhomogeneities were corrected using the data-driven approach UNICORT (Weiskopf et al., 2011) without correction for imperfect spoiling. An in-house MATLAB script (Version 9.7.0.1190202 (R2019b)) was used for the calculation of synT_1_-w images. According to the MPRAGE signal equation, the signal intensity (S) without undesired B_1_^+^/B_1_^-^effects was calculated assuming linear k-space sampling with centered main echo (Deichmann et al., 2000;Nöth et al., 2015;Schading, Seif, et al., 2023):

$$S=PD\cdot \sin\left(\alpha \right)\cdot \exp\left(-TE\cdot R_{2}^{\;*}\right)\cdot Q$$ (1)

Where*Q*is given by:

$$\frac{E_{4}\cdot \left(1-2\cdot E_{1}+E_{1}\cdot E_{2}\right)+T_{1}^{\;*}\cdot R_{1}\cdot \left(1+E_{1}\cdot E_{2}\cdot E_{3}-E_{1}\cdot E_{2}\cdot E_{4}-E_{4}\right)}{1+E_{1}\cdot E_{2}\cdot E_{3}}$$ (2)

With the parameters:

$$T_{1}^{\;*}={\left(R_{1}-\frac{1}{ES}\cdot \ln\left(\cos\left(\alpha \right)\right)\right)}^{-1}$$ (3)

$$E_{1}=\text{exp}\left(-\left(TI-\frac{\tau }{2}\right)\cdot R_{1}\right)$$ (4)

$$E_{2}=\text{exp}\left(-TD\cdot R_{1}\right)$$ (5)

$$E_{3}=\exp\left(-\frac{\tau }{T_{1}^{\;*}}\right)$$ (6)

$$E_{4}=\exp\left(-\frac{\tau }{2\cdot T_{1}^{\;*}}\right)$$ (7)

Where*α*= flip angle,*TE*= echo time,*TR*= repetition time,*TI*= inversion time,*ES*= echo spacing,*TD*= delay time, and*τ*= readout duration of the reconstructed synT_1_-w image.*TD*and*τ*can be derived as follows:

τ=n·ES (8)

$$TD=TR-\left(TI-\frac{\tau }{2}\right)-\tau$$ (9)

Where*n*denotes the number of sagittal partitions of the simulated MPRAGE acquisition.

### 2.5 Image processing and spinal cord segmentation

MPRAGE and synT_1_-w images were segmented and co-registered using the Spinal Cord Toolbox (De Leener et al., 2017) as follows: the images were automatically segmented with the*sct_deepseg_sc*algorithm (Gros et al., 2019) for obtaining binary masks of the spinal cord. These binary masks were carefully inspected and manually corrected if necessary. In case of extensive susceptibility and/or motion artifacts, the respective image or vertebral level was excluded from further analysis. The final sample size was 89.5% (C1), 87.4% (C2), and 79.1% (C3) of the total sample size. 6.8% of the data was excluded due to artifacts on MPRAGE and 10.6% on synT_1_-w images. Following segmentation, vertebral levels C1-C3 were semi-automatically identified and the MPRAGE and synT_1_-w images were co-registered to the MNI-Poly-AMU template (De Leener et al., 2018). Finally, the average CSA was obtained from the segmentations in every subject’s native space across vertebral levels C1-C3 separately, correcting for the angle between the cord’s centerline and the axial slices.

### 2.6 Optimization of synT _1_ -w parameters

To improve the accuracy of synT_1_-w images and to reduce the bias in CSA estimates between MPRAGE and synT_1_-w, the following parameters were optimized by a grid search within these ranges: α = 6–14°, TI = 400–1000 ms, TR = 1000–2500 ms, TE = 0–10 ms, and ES = 5–16 ms. For this optimization the first available synT_1_-w image from all healthy controls (n = 21) and the corresponding MPRAGE image were used. After reconstruction of the synT_1_-w image with the respective combination of parameters and extraction of the median CSA across C1-C3, the difference of CSA with respect to MPRAGE was calculated. The parameter combination resulting in the lowest absolute difference in CSA between synT_1_-w and MPRAGE was selected and applied to the remaining data set for further analysis.

### 2.7 Accuracy of CSA estimates from synT _1_ -w

All statistical analyses were performed in RStudio (Version 4.0.5). The accuracy of using synT_1_-w images in comparison to the reference standard MPRAGE for measuring the CSA of the upper cervical cord was assessed using the Bland-Altman method (Bland & Altman, 1986). The difference in CSA calculated from both segmentations (MPRAGE and synT_1_-w) was plotted against their average CSA. One data point corresponds to the CSA of one subject at one time point across vertebral levels C1-C3. The bias, which is a measure of accuracy, represents the average of the individual differences between MPRAGE and synT_1_-w. The upper and lower limits of agreement encompass 95% of the individual differences between MPRAGE and synT_1_-w.

The Bland-Altman analysis was conducted for the whole remaining data set, excluding the 21 MRI (synT_1_-w and MPRAGE) that were used for optimization. Moreover, the influence of a pathology (i.e., spinal cord injury) was assessed by running the Bland-Altman analysis separately for healthy controls and SCI patients. To assess whether the bias in both groups was statistically equivalent/similar, a Bayesian independent-samples T-test was conducted. For interpretation of the Bayes factor, the scale proposed by Harold Jeffreys was used (Jeffreys, 1961). A Bayes factor > 3 showed substantial evidence for the alternative hypothesis (i.e., the bias between healthy controls and SCI patients is different), while a Bayes factor < 0.33 was interpreted as substantial evidence for the null hypothesis (i.e., the bias between healthy controls and SCI patients is equivalent).

### 2.8 Precision of CSA measurements from synT _1_ -w

Given the focus of this study on optimizing the accuracy of CSA measurements derived from synT_1_-w images, it is important to assess whether an improvement in accuracy inadvertently reduces the precision of the measurements. To assess this concern, the standard deviation across repeated measurements (i.e., over time) was calculated and compared between those synT_1_-w images reconstructed using the new parameter settings and those from the previous study (Schading, Seif, et al., 2023). Only data from healthy controls were considered for this analysis, as their CSA is expected to remain relatively stable over the 2-year observation period.

### 2.9 Cord atrophy estimation based on MPRAGE and synT _1_ -w

For tracking changes in CSA over time and comparing MPRAGE with synT_1_-w estimates, the same linear mixed-effects model was created for CSA obtained from MPRAGE and from synT_1_-w. The model included vertebral levels (3 levels: C1-C3), time since injury/enrollment (5 time points) with a linear and a quadratic term to model deceleration effects, and their interaction with group (2 groups: healthy controls and SCI patients) as fixed effects. Random effects were the individual intercept and slope of time since injury and vertebral level, as well as the quadratic term of time since injury for every subject. Additionally, the model was corrected for the interaction of age with time since injury to account for aging-related changes (Papinutto et al., 2020), since there was a significant age difference between healthy controls and SCI patients, as well as for the MR scanner type due to the update over the course of the study.

Based on this model, the linear effects over time (i.e., linear atrophy rates), the quadratic effects (i.e., deceleration of atrophy), and the mean estimated CSA across C1-C3 at baseline and the 2-year follow-up were assessed and compared between healthy controls and SCI patients. These estimates for both groups and the difference between both groups were compared between the MPRAGE model and the synT_1_-w model. A significance threshold of p < 0.05 and Tukey’s correction for multiple comparison were used.

Moreover, a sample size estimation was conducted to calculate the required sample size for a hypothetical treatment for detecting a reduction of cord atrophy at the 2-year follow-up in spinal cord injury patients with 80% power and a 5% significance threshold. This sample size estimation was based on a two-group trial (baseline-adjusted comparison of the means, analysis of covariance) with one intervention group who underwent the hypothetical treatment and one control group of spinal cord injury patients who received the current standard of care. The treatment effect was determined using the CSA of healthy controls as a reference value, where the difference in CSA between spinal cord injury patients and healthy controls at 2 years after injury was calculated for different treatment effects ranging from 20–80%. To illustrate this, a 100% treatment effect indicates that this SCI patient cohort would not show CSA atrophy 2 years after injury, while a 0% treatment effect corresponds to the same CSA atrophy detectable in the treatment as in the control SCI group. The Pearson correlation coefficient between the CSA at baseline and the 2-year follow-up was calculated from the available data, and the sample size estimation was conducted in the range from the lowest to the highest correlation to cover a range of plausible values.

## 3 Results

### 3.1 Optimization of synT _1_ -w parameters

**Fig. 1.**
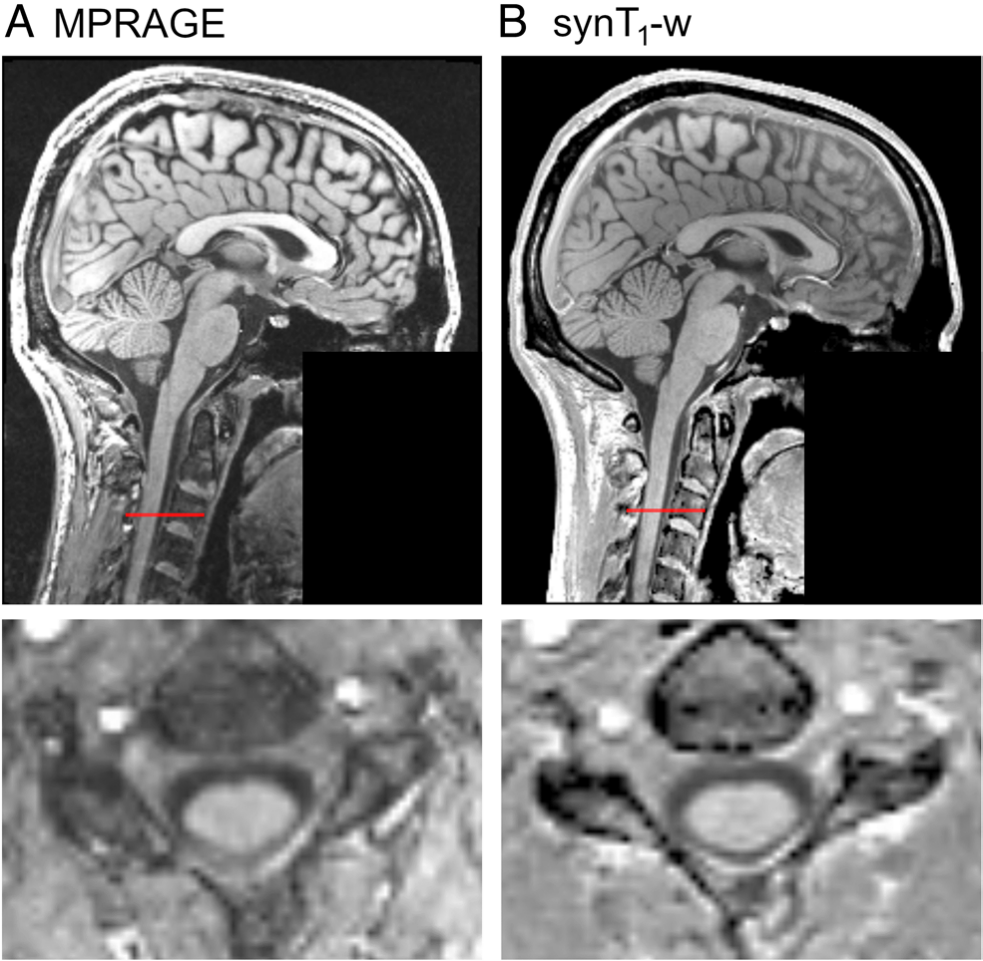
Illustration of MPRAGE (A) and the corresponding synT_1_-w (B) of a healthy participant. The synT_1_-w was reconstructed using the optimized image parameters that resulted in the minimal difference in CSA between MPRAGE and synT_1_-w. The bottom row shows an axial view of the respective image at vertebral body C3 (the red line indicates the position of the axial slice). Note that facial structures were removed from the images for data protection considerations.

The minimal difference of average CSA across C1-C3 between MPRAGE and synT_1_-w was obtained with the parameters of α = 14°, TI = 900 ms, TR = 1400 ms, TE = 0 ms, and ES = 14 ms.Figure 1shows a representative synT_1_-w image using these parameters as well as the corresponding MPRAGE image for illustration. The mean difference in CSA between MPRAGE and synT_1_-w was -0.07 mm^2^(-0.11%) for these parameters (SupplementaryFig. S1).

### 3.2 Accuracy of CSA estimates from synT _1_ -w

**Fig. 2.**
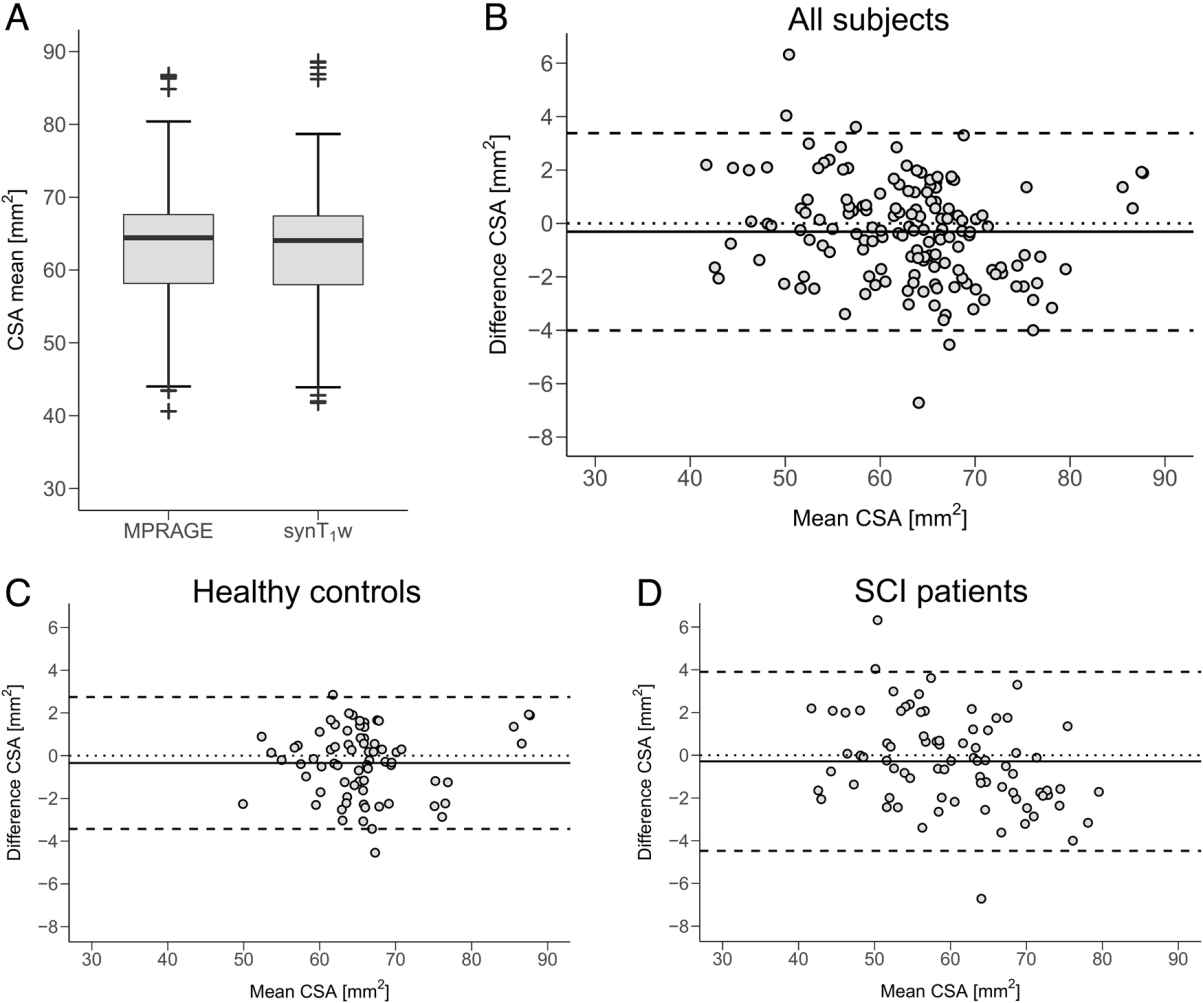
Average spinal cord cross-sectional area (CSA) across C1-C3 for MPRAGE and synT_1_-w (A). Bland-Altman plot for CSA derived from MPRAGE and synT_1_-w for all subjects (B) and for healthy controls (C) and spinal cord injury (SCI) patients (D) separately.

When comparing the CSA from the remaining dataset (i.e., without the 21 scans from optimization), the average CSA for MPRAGE was 63.02 ± 9.11 mm^2^and for synT_1_-w 62.71 ± 8.70 mm^2^(Fig. 2A). In the Bland-Altman analysis, the corresponding bias of CSA obtained from synT_1_-w was -0.31 mm^2^(-0.5%) with a lower limit of agreement of -4.01 mm^2^(-6.4%) and an upper limit of agreement of 3.38 mm^2^(5.4%) (Fig. 2B). For investigating whether the presence of a spinal cord trauma has an impact on the accuracy of estimating CSA based on synT_1_-w images, the Bland-Altman analysis was conducted for healthy controls and SCI patients separately (Fig. 2C&D). Here, the bias of controls was -0.34 mm^2^(-0.5%) with limits of agreement of -3.43 mm^2^(-5.2%) and 2.74 mm^2^(4.2%), while SCI patients showed a bias of -0.29 mm^2^(-0.5%) with limits of agreement of -4.70 mm^2^(-7.4%) and 4.17 mm^2^(6.5%), respectively. The Bayes factor for the comparison between the bias of controls and patients was smaller than 0.33 (BF_10_= 0.178), providing evidence for the null hypothesis (i.e., both groups show equivalent biases).

### 3.3 Precision of CSA estimates from synT _1_ -w

Comparing the precision of the CSA measurements of healthy controls derived from synT_1_-w images of the current study and the previous study, the average standard deviation was slightly higher (current study [mean ± SD]: 1.41 ± 0.52 mm^2^, previous study: 1.33 ± 0.55 mm^2^).

### 3.4 Cord atrophy estimation based on MPRAGE and synT _1_ -w

**Fig. 3.**
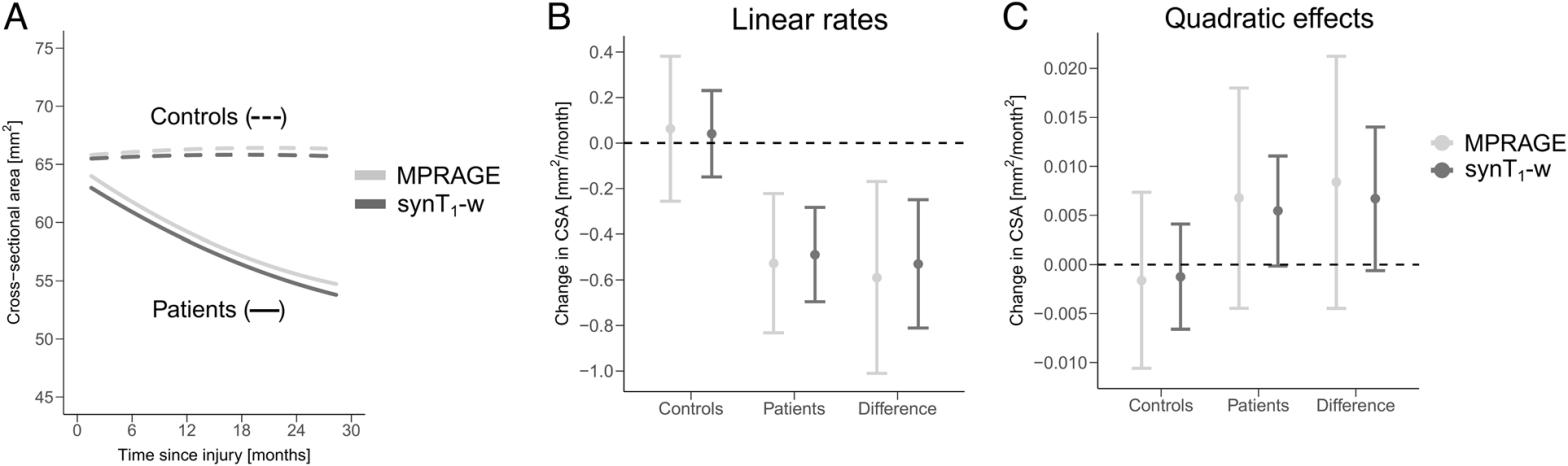
Change in CSA over 2 years for healthy controls (dashed line) and SCI patients (solid line) estimated based on MPRAGE (light gray) and synT_1_-w (dark gray) across C1-C3 (A). Linear effects (i.e., linear atrophy rates) of controls, SCI patients and the difference between both groups estimated from linear mixed-effects models (B). Quadratic effects (i.e., deceleration of atrophy) of controls, SCI patients and the difference between both groups estimated from linear mixed-effects models (C).

Figure 3Ashows the linear mixed-effects models for tracking cord atrophy over 2 years following SCI. The minor remaining bias between MPRAGE and synT_1_-w from previous Bland-Altman analysis is observable in the model as a general offset between both curves from MPRAGE and synT_1_-w.

The linear atrophy rates and quadratic effects are plotted inFigure 3B&Cand reported inTable 1. Both models (MPRAGE and synT_1_-w) resulted in similar estimates for linear and quadratic effects of CSA changes over 2 years following SCI. The estimates were comparable for healthy controls and SCI patients. A significant difference in the linear atrophy rate of CSA between SCI patients and healthy controls was detected using both models (MPRAGE: -0.590 mm^2^/month, p = 0.027; synT_1_-w: -0.530 mm^2^/month, p = 0.0003).

**Table 1.** Linear rates of CSA atrophy averaged across C1-C3 for MPRAGE and synT_1_-w estimated from linear mixed-effects models.

Cross-sectional area linear rate							
	Controls		Patients		Patients – controls		
Protocol	Rate [mm ^2^ /month]	95% CI [mm ^2^ /month]	Rate [mm ^2^ /month]	95% CI [mm ^2^ /month]	Rate [mm ^2^ /month]	95% CI [mm ^2^ /month]	p-value
MPRAGE	0.063	-0.256 to 0.382	-0.527	-0.832 to -0.222	-0.590	-1.011 to -0.170	0.027
synT _1_ -w	0.041	-0.149 to 0.231	-0.489	-0.695 to -0.283	-0.530	-0.811 to -0.249	0.0003
Difference	-0.022		0.038		0.060		

Cross-sectional area quadratic effect							
	Controls		Patients		Patients – controls		
Protocol	Rate [mm ^2^ /month ^2^ ]	95% CI [mm ^2^ /month ^2^ ]	Rate [mm ^2^ /month ^2^ ]	95% CI [mm ^2^ /month ^2^ ]	Rate [mm ^2^ /month ^2^ ]	95% CI [mm ^2^ /month ^2^ ]	p-value
MPRAGE	-0.0016	-0.0106 to 0.0073	0.0068	-0.0045 to 0.0180	0.0084	-0.0045 to 0.0213	0.10
synT _1_ -w	-0.0012	-0.0066 to 0.0041	0.0055	-0.0001 to 0.0111	0.0067	-0.0006 to 0.0140	0.072
Difference	0.0004		-0.0013		-0.0017		

CSA: cross-sectional area, MPRAGE: Magnetization Prepared Rapid Acquisition Gradient-Echo image, synT_1_-w: synthetic T_1_-w image.

The average estimates of CSA at baseline and the 2-year follow-up as well as the difference between healthy controls and SCI patients are depicted inFigure 4and reported inTable 2. Both, average estimates of CSA for healthy controls and SCI patients separately as well as the difference between both groups were comparable between MPRAGE and synT_1_-w. While in both models no significant differences in CSA between controls and SCI patients were detected at baseline (MPRAGE: -1.81 mm^2^, p = 0.34; synT_1_-w: -2.51 mm^2^, p = 0.20), CSA was significantly reduced in SCI patients at the 2-year follow-up with similar magnitudes in MPRAGE (-11.59 mm^2^, p = 0.0007) and synT_1_-w (-11.89 mm^2^, p = 0.0001).

**Fig. 4.**
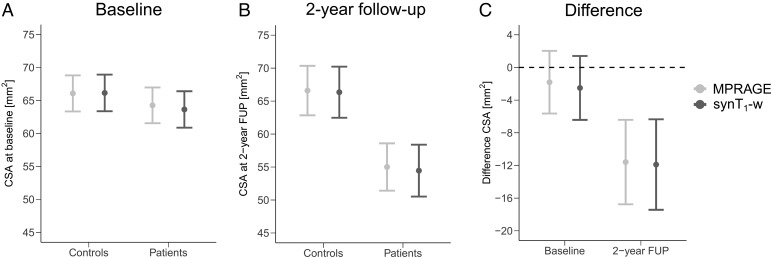
Average CSA at baseline (A) and 2-year follow up (B) for controls, SCI patients and the difference between both groups (C) estimated from linear mixed-effects models. MPRAGE - light gray; synT_1_-w - dark gray.

**Table 2.** CSA averaged across C1-C3 for MPRAGE and synT_1_-w at baseline and 2-year follow-up estimated from linear mixed-effects models.

Average cross-sectional area								
		Controls		Patients		Patients – controls		
Time point	Protocol	CSA [mm ^2^ ]	95% CI [mm ^2^ ]	CSA [mm ^2^ ]	95% CI [mm ^2^ ]	CSA [mm ^2^ ]	95% CI [mm ^2^ ]	p-value
Baseline	MPRAGE	66.08	63.34 to 68.81	64.27	61.56 to 66.98	-1.81	-5.65 to 2.03	0.34
	synT _1_ -w	66.15	63.37 to 68.93	63.64	60.88 to 66.41	-2.51	-6.42 to 1.40	0.20
	Difference	0.07		-0.63		-0.70		
2-year FUP	MPRAGE	66.60	62.84 to 70.35	55.01	51.42 to 58.60	-11.59	-16.76 to -6.42	0.0007
	synT _1_ -w	66.35	62.48 to 70.22	54.45	50.52 to 58.39	-11.89	-17.43 to -6.35	0.0001
	Difference	-0.24		-0.55		-0.30		

CSA: cross-sectional cord area, FUP: follow-up, MPRAGE: Magnetization Prepared Rapid Acquisition Gradient-Echo image, synT_1_-w: synthetic T_1_-w image

**Fig. 5.**
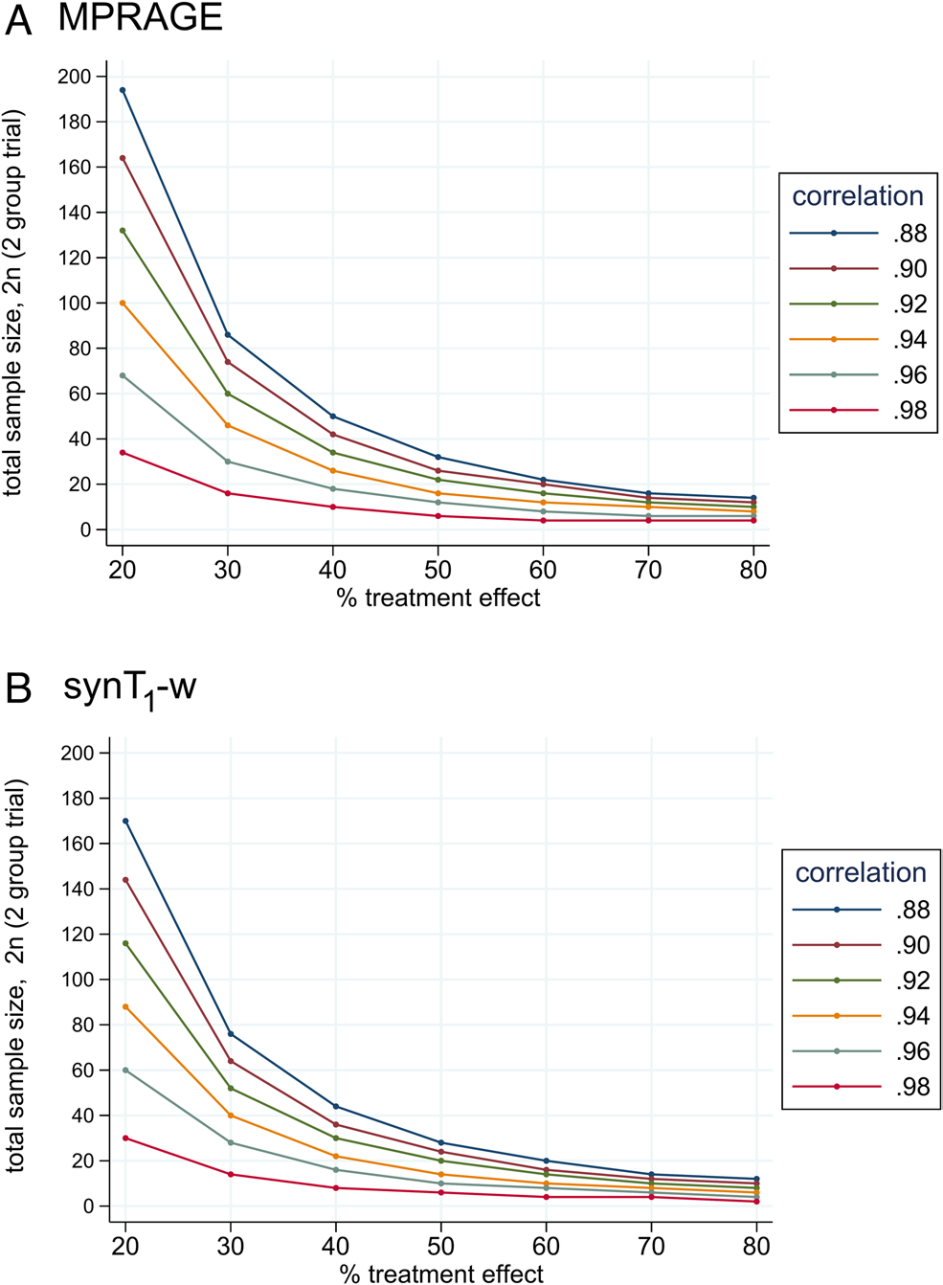
Sample size estimation for detecting effects of a hypothetical treatment as a reduction of the CSA difference at the 2-year follow-up with 80% power and a 5% significance threshold for MPRAGE (A) and synT_1_-w (B), based on a two-group trial (baseline-adjusted comparison of the means, analysis of covariance) with one SCI patient group receiving the hypothetical treatment and one SCI patient group receiving the current standard of care. A treatment effect of 100% would indicate that this SCI patient cohort would not show CSA atrophy 2 years after injury, while a 0% treatment effect corresponds to the same CSA atrophy detectable in the treatment as in the control SCI group. The reported correlation coefficients represent the Pearson correlation between the CSA at baseline and the 2-year follow-up.

The sample size estimation showed that the required sample size for detecting treatment effects with a power of 80% and a significance threshold of 5% can be reduced by 13.5% (±8.7%) when using synT_1_-w instead of MPRAGE (Fig. 5& SupplementaryTable S3). For example, given a Pearson correlation between baseline and the 2-year follow-up of 0.94, which lies approximately in the middle of the observed range of correlation coefficients, to detect a 30% effect on CSA (i.e., reduction of the difference in CSA between control and treatment group by 30%), 40 participants are required when using synT_1_-w, while 46 participants are necessary for MPRAGE. This corresponds to a reduction of 13.0% in the study population when using synT_1_-w instead of MPRAGE.

## 4 Discussion

This study improved the accuracy of macrostructural measures of the spinal cord CSA derived from synthetic T_1_-w MRI by minimizing the difference to the reference standard MPRAGE compared to previous approaches (Schading, Seif, et al., 2023). Moreover, reliable estimates of cord atrophy following SCI were obtained from synT_1_-w MRI. These findings may have significant implications for the planning of future MRI studies, as they demonstrate that after calibration of the synT_1_-w parameters, reliable and accurate estimates of CSA are obtained from synT_1_-w images. This suggests that synthetic MRI can replace MPRAGE in clinical studies, reducing total acquisition time by approximately 10 minutes—depending on the acquisition protocol. Moreover, a smaller number of participants is required when using the synT_1_-w approach instead of MPRAGE, which can help to further increase the efficiency of clinical trials.

### 4.1 Optimization of synT _1_ -w parameters

The final sequence parameters for the reconstruction of the synT_1_-w image slightly differed from the MPRAGE MRI parameters. Essentially, a conventional MPRAGE acquisition using these synT_1_-w parameter settings would not be feasible due to the timing of the pulses as dictated by TI, TR, and echo spacing of the optimized synT_1_-w settings. However, since the reconstruction of the synT_1_-w images is not limited by pulse timing restraints, the contrast of the reconstructed images can be adapted very flexibly, pointing at one of the advantages of synthetic MRI.

### 4.2 Accuracy of CSA estimates from synT _1_ -w

In clinical studies, both high accuracy and high precision of measurements are fundamental. Previously, we determined the test-retest repeatability of synT_1_-w MRI for measuring the upper cervical cord CSA and optimized the parameters of synT_1_-w with regard to precision (Schading, Seif, et al., 2023). We showed high repeatability of CSA measurements based on synT_1_-w images both within the same scanner (i.e., intra-site) and between different scanner systems (i.e., inter-site), which is a crucial factor for planning clinical studies, in particular multi-center studies. However, when directly comparing the estimates of CSA derived from synT_1_-w and the reference standard MPRAGE, synT_1_-w showed a remaining systemic bias of -1.59 mm^2^(-2.5%), which hampers the comparability with studies using acquired MPRAGE images for assessing the cervical cord CSA. Using the optimized synT_1_-w images in this study, the remaining bias decreased to -0.31 mm^2^(-0.5%), which greatly enhances the direct comparability with different studies. Considering the in-plane resolution of the MPRAGE and synT_1_-w images of 1 x 1 mm^2^, this bias of -0.31 mm^2^is remarkable, since it indicates that on average across all subjects the error was approximately 1/3 of the voxel size. However, as this study aimed to maximize the accuracy of CSA measurements, there was a theoretical concern that this optimization might adversely impact precision compared to synT_1_-w images previously optimized for precision. When comparing the standard deviations of the CSA measurements between the current and our previous study (Schading, Seif, et al., 2023), a slight decrease in precision was observed, indicating that parameter optimization for accuracy may, indeed, lead to a reduction in precision, suggesting that optimization of the parameters with respect to accuracy comes at the cost of a reduction in precision. However, the difference in precision was only marginal. Thus, while optimization for accuracy may come at a modest cost to precision, it remains reasonable to prioritize harmonization of measurements to minimize bias, particularly in the context of multi-center studies. In a multi-center setting, the bias will not only reduce the accuracy of the measurements but also reduce the overall precision, since it increases variability of measurements across sites.

Given that SCI patients, in particular patients with lesions in the cervical cord, show pathologic alterations as well as potential susceptibility artifacts due to the presence of metallic implants and increased motion artifacts due to spasticity (Andresen et al., 2016;DiPiro et al., 2018;Freund et al., 2019), the question remains, whether the presence of a pathology affects the accuracy of CSA estimates based on synT_1_-w images. This could impair the validity of using synT_1_-w in clinical studies for tracking neurodegeneration in SCI patients. Therefore, we estimated the bias of synT_1_-w for healthy controls and SCI patients separately and compared it between both groups. Using Bayesian statistics, we found evidence in support of the null hypothesis stating that the bias in healthy controls and SCI patients is equivalent, meaning that the presence of a spinal cord injury does not systematically impair the validity of CSA measurements based on synT_1_-w images. Thus, we conclude that synT_1_-w images provide accurate estimates of CSA for both healthy controls and SCI patients, which corroborates the applicability of synT_1_-w for tracking SCI-induced cord atrophy.

### 4.3 Cord atrophy estimation based on MPRAGE and synT _1_ -w

For validating the application of the optimized synT_1_-w images in a clinical study, we could show that atrophy rates (i.e., change/time) of the cervical cord CSA following SCI could be estimated accurately. Additionally, the average CSA at several time points after injury was similar between MPRAGE and synT_1_-w as well. The CSA reduction in SCI patients compared to controls (MPRAGE: -11.59 mm^2^, 95% CI -16.76 to -6.41 mm^2^; synT1-w: -11.89 mm^2^, 95% CI -17.43 to -6.35 mm^2^) was in line with previously reported atrophy from a meta-analysis (-13.5 mm^2^, 95% CI -16.6 to -10.5 mm^2^) (Trolle et al., 2023).

The high accuracy of cross-sectional comparisons indicates that synT_1_-w-derived measures of a single measurement instead of repetitive assessments can be investigated as potential predictive markers. This could help for establishing early prognostic imaging biomarkers and could facilitate their implementation in clinical routine (Seif, Curt, et al., 2018).

Another important observation pertains to the variance of the estimates of atrophy. When comparing synT_1_-w with MPRAGE, the estimates of atrophy showed smaller standard errors in the synT_1_-w models, indicating that the change of CSA over time could be estimated more reliably due to lower variance. This might be related to a higher signal-to-noise ratio of the synT_1_-w images compared to MPRAGE (SupplementaryFig. S2). However, since acquisition time plays a major role for the signal-to-noise ratio, it must be highlighted that the acquisition time for the MPM protocol, which underlies the reconstructed synT_1_-w image, was approximately 23 minutes, while the MPRAGE sequence was acquired within 9 minutes. Another factor reducing variability might be that the synT_1_-w images were reconstructed based on quantitative MRI with quantitative parameters that are more comparable across different MR scanners and time points (Weiskopf et al., 2021). This reduction in variability can have important implications for prospective clinical trials, since higher statistical power can be achieved with lower variance in the outcome measures. Our sample size estimation confirmed this hypothesis as it showed that when using synT_1_-w instead of MPRAGE, the required sample size can be reduced by approximately 13.5%. This increase in efficiency should certainly be considered when planning clinical studies.

These results show that after initial calibration of the synT_1_-w parameters using acquired MPRAGE, synT_1_-w images based on quantitative maps from the MPM protocol (Weiskopf et al., 2013) provide accurate and valid estimates of cervical cord CSA and atrophy following SCI. Crucially, the presence of a pathology (i.e., spinal cord injury) with its consequences such as metal and motion artifacts did not impair the estimation of cord atrophy in SCI patients compared to healthy controls. Therefore, synT_1_-w MRI may be considered as an alternative to acquiring an additional MPRAGE for assessing changes in the spinal cord’s macrostructure, which reduces the acquisition time of long imaging protocols. In particular, the increase in accuracy of synthetic MRI post-harmonization may be beneficial for multi-center studies by reducing potential center biases and inter-center variation. Moreover, synT_1_-w allows to reduce the required sample size in prospective clinical trials. This improves both patient comfort and the efficiency of study protocols and paves the way for applying synthetic MRI in clinical studies.

### 4.4 Limitations

Over the course of the study, the scanner was upgraded to a newer version with a change of certain software and hardware elements. However, this upgrade affected both MPRAGE and synT_1_-w images equally. Thus, the comparison between MPRAGE and synT_1_-w images, which is the main focus of this study, is not expected to be greatly affected. Moreover, the sample size for assessing atrophy in the cervical cord was relatively small and some scans had to be excluded due to motion and/or susceptibility artifacts. However, the main aim of this study was to directly compare synT_1_-w with the reference standard MPRAGE. Since the final data set comprised exactly the same scans for both synT_1_-w and MPRAGE, a valid comparison between both modalities was possible. We also note that we estimated the accuracy by comparisons to MPRAGE acquisitions. It is a pragmatic approach, since MPRAGE is widely used and was validated against phantoms (Freund et al., 2010), but it was not directly validated by comparing to neuroanatomical measures. In practice, it is of less relevance, since primarily the harmonization of metrics is central for multi-center clinical trials and the absolute bias is expected to be relatively small based on previous studies. Finally, the study cohort included patients with lesions at various spinal levels (C3–T11), potentially introducing variability in atrophy measures in the upper cervical cord and, consequently, affecting sample size estimations. However, the main purpose of this study was to assess and compare the required sample sizes between acquired and synthetic MRI. Notably the patient cohorts for both sample size calculations (MPRAGE and synT_1_-w) remained the same. As a result, valid comparisons can be made between both methods.

## 5 Conclusion

This study improved the accuracy of CSA measurements (i.e., minimizing the difference in CSA between synT_1_-w and the reference standard MPRAGE) and demonstrated that the parameters of synT_1_-w could be further optimized. Crucially, these optimized synT_1_-w images allowed for reliable tracking of cervical cord atrophy over 2 years following SCI with accurate estimates of atrophy rates and average CSA for both healthy controls and SCI patients. This suggests that after calibration of the synT_1_-w parameters with MPRAGE, the acquisition time of current study protocols may be reduced by approximately 10 minutes (Freund et al., 2013), which facilitates the potential use in clinical studies. Moreover, fewer participants are required to detect a potential treatment effect in a clinical trial setting when using synthetic MRI.

## Supplementary Material

Supplementary Material

## Data Availability

The authors certify they have documented all data, methods, and materials used to conduct the research presented. Anonymized data pertaining to the research presented will be available by request from investigators.
