# Supplementary material for: Optimization of cervical cord synthetic T 1 -weighted MRI for enhancing clinical application in neurodegenerative spinal cord disorders
Source: Imaging Neurosci (Camb). 2024 Jul 15;2:imag-2-00225. doi: 10.1162/imag_a_00225 (PMC12272268; doi:10.1162/imag_a_00225)
Supplement: Supplementary Material [file imag_a_00225-supp.pdf]

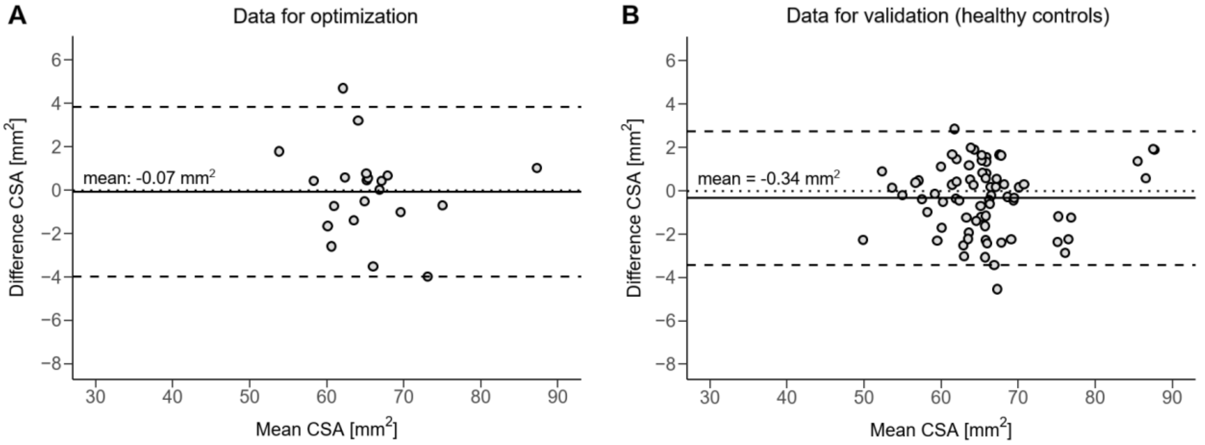

Supplementary Figure S1. Comparison of the bias between data set used for optimization (A) and validation (B) (only healthy controls). A Bayesian independent samples t-test showed evidence for the null hypothesis that the biases of both groups are equivalent (BF_{10} = 0.302), suggesting that the reconstruction parameters work well on both the optimization and the validation cohort (i.e. no overfitting).

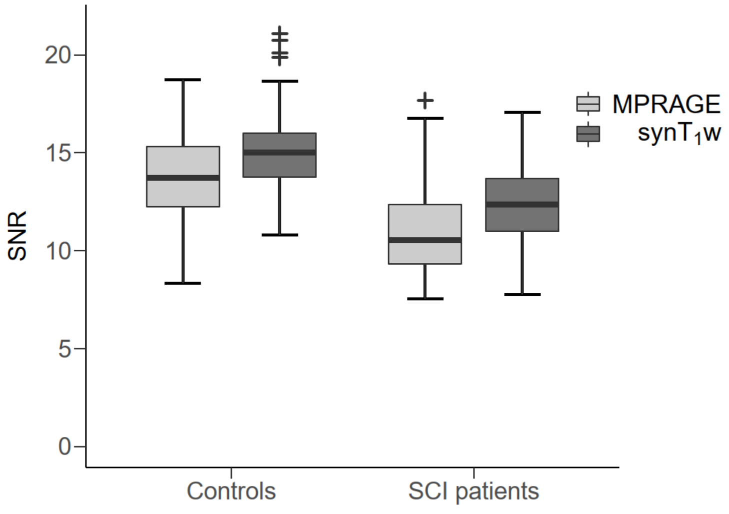

Supplementary Figure S2. Comparison of the signal-to-noise ratio (SNR) between MPRAGE and synT$_1$-w. The average SNR for synT$_1$-w (across controls and SCI patients) was significantly higher than for MPRAGE (synT$_1$-w: 13.87, MPRAGE: 12.60, $F = 31.88$, $p < 0.0001$, *analysis of variance [ANOVA]*).

Supplementary Table S3. Sample size estimation for MPRAGE and synT$_1$-w

Correlation	Treatment effect [%]	Sample size (treatment + control)	
		MPRAGE	synT$_1$-w
0.88	20	194	170
	30	86	76
	40	50	44
	50	32	28
	60	22	20
	70	16	14
	80	14	12
0.90	20	164	144
	30	74	64
	40	42	36
	50	26	24
	60	20	16
	70	14	12
	80	12	10
0.92	20	132	116
	30	60	52
	40	34	30
	50	22	20
	60	16	14
	70	12	10
	80	10	8
0.94	20	100	88
	30	46	40
	40	26	22
	50	16	14
	60	12	10
	70	10	8
	80	8	6
0.96	20	68	60
	30	30	28
	40	18	16
	50	12	10
	60	8	8
	70	6	6
	80	6	4
0.98	20	34	30
	30	16	14
	40	10	8
	50	6	6
	60	4	4
	70	4	4
	80	4	2
